# An Experimental Anodized and Low-Pressure Oxygen Plasma-Treated Titanium Dental Implant Surface—Preliminary Report

**DOI:** 10.3390/ijms24043603

**Published:** 2023-02-10

**Authors:** Jakub Hadzik, Kamil Jurczyszyn, Tomasz Gębarowski, Andrzej Trytek, Tomasz Gedrange, Marcin Kozakiewicz, Marzena Dominiak, Paweł Kubasiewicz-Ross, Anna Trzcionka-Szajna, Ernest Szajna, Wojciech Simka

**Affiliations:** 1 Department of Dental Surgery Medical, Faculty of Medicine and Dentistry, University of Wrocław, 50-425 Wrocław, Poland; 2 Department of Biostructure and Animal Physiology, Wrocław University of Environmental and Life Sciences, 51-631 Wroclaw, Poland; 3 Faculty of Mechanics and Technology, Rzeszow University of Technology, 37-450 Stalowa Wola, Poland; 4 Division of Orthodontics, Technical University Dresden, 01307 Dresden, Germany; 5 Department of Maxillofacial Surgery, Faculty of Military Medicine, Medical University of Łódź, 90-419 Łódź, Poland; 6 NanoPrime, 39-200 Dębica, Poland; 7 WEA Techlab, 41-301 Dąbrowa Górnicza, Poland; 8 Faculty of Chemistry, Silesian University of Technology, 44-100 Gliwice, Poland

**Keywords:** fractal dimension analysis, implant topography, oxygen plasma, plasma electrolytic oxidation, texture analysis

## Abstract

Chemical composition and physical parameters of the implant surface, such as roughness, regulate the cellular response leading to implant bone osseointegration. Possible implant surface modifications include anodization or the plasma electrolytic oxidation (PEO) treatment process that produces a thick and dense oxide coating superior to normal anodic oxidation. Experimental modifications with Plasma Electrolytic Oxidation (PEO) titanium and titanium alloy Ti6Al4V plates and PEO additionally treated with low-pressure oxygen plasma (PEO-S) were used in this study to evaluate their physical and chemical properties. Cytotoxicity of experimental titanium samples as well as cell adhesion to their surface were assessed using normal human dermal fibroblasts (NHDF) or L929 cell line. Moreover, the surface roughness, fractal dimension analysis, and texture analysis were calculated. Samples after surface treatment have substantially improved properties compared to the reference SLA (sandblasted and acid-etched) surface. The surface roughness (Sa) was 0.59–2.38 µm, and none of the tested surfaces had cytotoxic effect on NHDF and L929 cell lines. A greater cell growth of NHDF was observed on the tested PEO and PEO-S samples compared to reference SLA sample titanium.

## 1. Introduction

Titanium (Ti) and titanium alloys have found their way into dentistry as a material for dental implants. Titanium is biologically inert, able to bond with osteoblasts, and has excellent biocompatibility [1]. Moreover, titanium shows excellent corrosion resistance due to titanium oxide, which forms a passive film on its surface. The titanium oxide (TiO_2_) film is typically around 3–10 nm, so it can stably stay on the Ti surface [2].

Implant surface chemical composition and physical surface topography parameters such as the roughness of dental implant surfaces have all been linked to biological regulation in cell interactions leading to osseointegration [3].

The physical, chemical, and biological assessment of the implant surface need to be performed before being approved for clinical trials. To compare the surface topography of different implants, appropriate mathematical and physical descriptive methods can be used. A typical physical description of the implant surface topography is described by the Ra, Rz, and Sa. The degree of roughness, marked as Ra (Average roughness), is an average measure of the deviation value of individual surface points in relation to the selected reference plane [4]. Rz is referred to as the maximum vertical roughness, and Sa is a parameter expressing the absolute value of the difference in height of each point compared to the arithmetic mean of the surface [4]. Sa is often used to describe implant surface roughness, and its values between 1 µm and 2 µm appear to be optimal for dental implants, however, the mechanisms behind an optimal bone response to this Sa value remain largely unknown [5]. Among the mathematical methods, a fractal dimension (FD) analysis can be listed together with the texture analysis (TA). FD analysis can be applied in the description of irregular or complex surfaces or shapes. Fractal dimension analysis (FDA) has been reported in surface testing of various dental materials; among them are xenogeneic bone substitutes, lithium disilicate-based crowns, zirconia dental implants, dental restorative composite, or orthodontic wires [6,7,8,9]. Since the fractal architecture concept is particularly interesting in surface and materials science, it also can be adapted to assess the surface of titanium dental implants. Texture analysis is used in dentistry or medicine to analyze images of computed tomography or X-ray [10,11,12,13]

Numerous implant surface modifications have been presented in the literature, most commonly used for dental implant sandblasting and etching with acid (SLA) and nanostructure-modified surfaces [14]. These modifications, by affecting the biological tissue response, undoubtedly affect both the possibility of earlier loading of the implant as well as shortening implant length [15]. Plasma electrolytic oxidation, used in our study, was used to improve the properties of medical implants. Plasma can increase the adhesiveness of the surface, therefore, can modify the surface cell adhesions.

The first aim of the study was to compare the reference SLA titanium surface to the plasma electrolytic oxidated (PEO) and PEO treated with low-pressure oxygen plasma (PEO-S) titanium plates. The second aim was to check if the texture and fractal dimension analysis can be used to evaluate dental implant surfaces with complex geometry.

The research null hypotheses were raised:

1. None of the experimental samples express cytotoxicity.
2. There is no difference between the cell growth and cell adherence of the experimental surface modified with plasma electrolytic oxidation (PEO) titanium plates and PEO treated with low-pressure oxygen plasma (PEO-S) compared to standard SLA surface.
3. There are no correlations between a fractal dimension (FD) and texture analysis (TA), nor between implant surface roughness, Sa, and cell growth.
4. There are no differences between evaluated surfaces in an aspect of FD and TA.

## 2. Results

**Figure 1:**
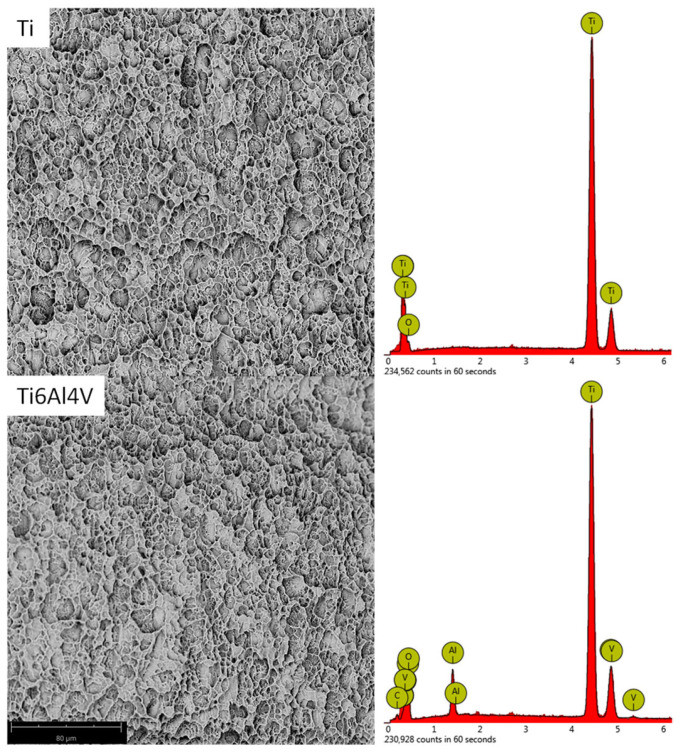
The SEM (scanning electron microscope) images and EDX (energy-dispersive X-ray spectrometer) spectra of Ti and Ti6Al4V samples after sandblasting and etching.

The titanium (Ti) and Ti6Al4V alloy have been sandblasted (Al_2_O_3_) and then etched to increase surface roughness. As a result of these processes, characteristic craters and pores formed on the surface (Figure 1). After the etching process, all embedded sand grains were removed, and the surface was chemically homogeneous (Figure 1). Only substrate elements (Ti or Ti, Al, and V) and oxygen were detected on the surface. The presence of oxygen is related to the naturally formed oxide layer on titanium and its alloys in the presence of air [16].

**Figure 2:**
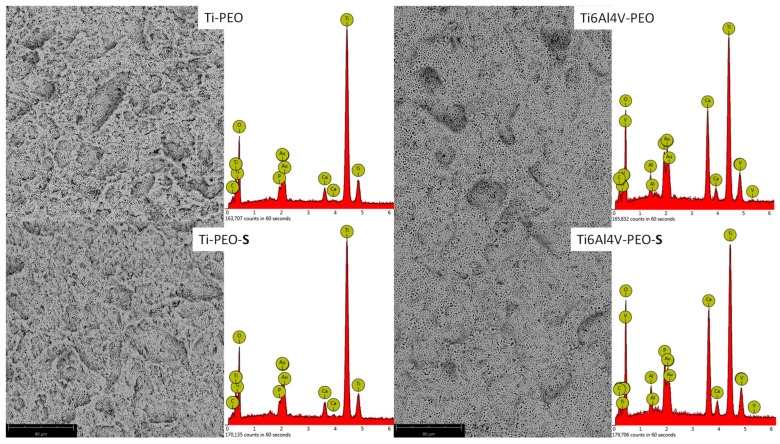
The SEM images of etched Ti and Ti6Al4V samples after the PEO process (Ti-PEO, Ti6Al4V-PEO) and after the PEO process with oxygen plasma treatment (Ti-PEO-S, Ti6Al4V-PEO-S).

The sandblasted and etched samples are subjected to the plasma electrochemical oxidation (PEO) process in a solution containing calcium and phosphorus compounds [17,18]. During this process, the natural oxide layer on the titanium is thickened. There is an electric discharge of the forming oxide layer, and plasma with a temperature of several thousand degrees Celsius is formed in the puncture channels [19]. This melts and then solidifies the oxide layer. At the same time, the components of the electrolyte, calcium, and phosphorus compounds are incorporated into it [20,21]. The surface morphology of the modified samples is typical for the PEO process (Figure 2). The SEM (scanning electron microscope) images show typical pores resulting from discharges. Analysis of the chemical composition of EDX (energy-dispersive X-ray spectrometer) confirmed the incorporation of calcium and phosphorus into the oxide layer, both in titanium and the Ti6Al4V alloy (Ti-PEO and Ti6Al4V-PEO samples; Figure 2 and Table 1). The EDX spectra also show peaks from the gold that was sputtered on the samples before the SEM observations. Calcium and phosphorus incorporated into the oxide layer are present in the phosphate form [22,23]. It can also be seen that more calcium was incorporated into the Ti6Al4V alloy than in the case of titanium. Additional treatment of the samples with oxygen plasma did not change the surface morphology of the samples (Ti-PEO-S and Ti6Al4V-PEO-S samples; Figure 2). However, their chemical composition changed slightly. In the case of modified titanium and alloy in the oxide layer, the oxygen content increased slightly—around 1% (Ti-PEO-S and Ti6Al4V-PEO-S samples; Table 1). This means that titanium and alloy were oxygenated, which may have a positive effect on their biological response [24].

### 2.1. Surface Roughness Outcomes

Sa average surface height deviation amplitude was highest for the Ti-PEO surface and reached 2.38 µm; the lowest value of Sa 0.59 µm was noted for the Ti6Al4V-PEO-S sample. Surface roughness (Sa) for all the examined surfaces is presented in Table 2.

### 2.2. Biological Analysis Outcomes

#### 2.2.1. In Vitro Cytotoxicity Assessment

**Figure 3:**
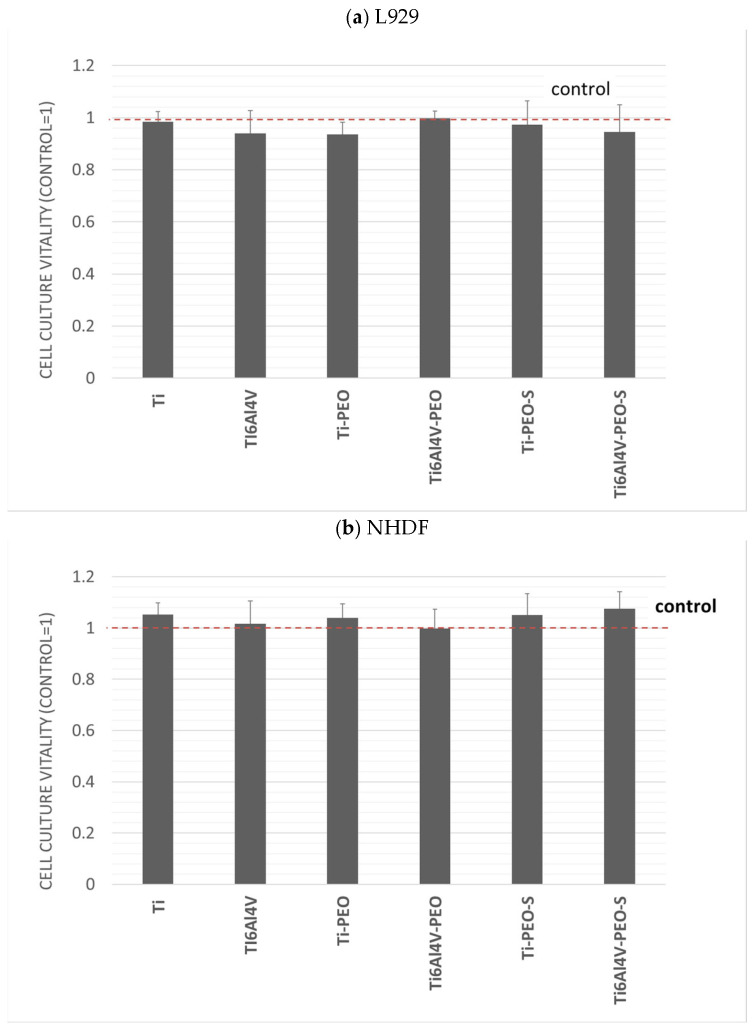
(**a**) cell culture vitality of the L929, and (**b**) cell culture vitality of the NHDF. Evaluation of the cytotoxicity of the tested biomaterials. Results are averages from 5 independent experiments, presented as a ratio of the value obtained in the test culture to the control. Red line—control. There was no statistically significant decrease in the viability of the culture compared to the control (*p* < 0.05).

The test was performed according to the protocol described in the standard for the cytotoxicity of medical devices. The study assessed the effect of extracts obtained from biomaterials on the vitality of two cell lines used for this purpose. No toxicity of the tested extracts was found. The results are shown in the graphs (Figure 3). No significant effect on the vitality of the tested cultures was found in the tests performed.

In the MTT assay, the L929 consensus reference line and the normal human dermal fibroblasts (NHDF) line were used. For both lines, cell morphology assessment was performed according to the guidelines described in the standard and the laboratory procedures. The assessment of cell morphology was performed by comparison with the specifications for the lines and control cultures. No cytopathic changes were observed in the cultures.

#### 2.2.2. Co-Culture of Cells with Test Materials

**Figure 4:**
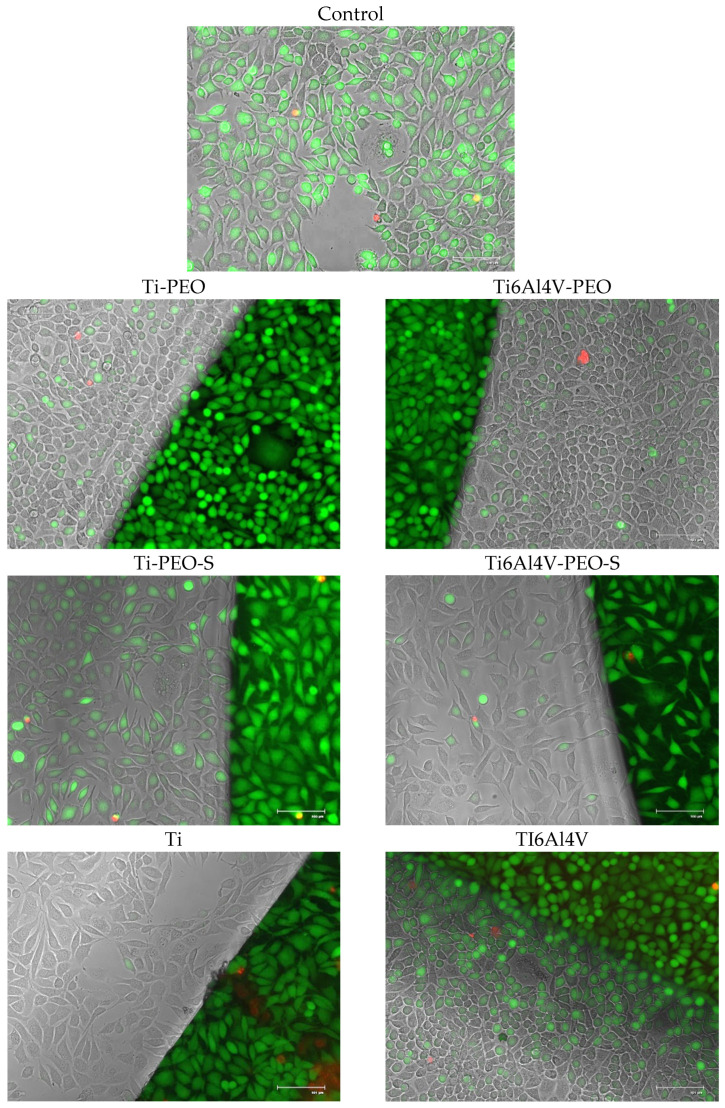
Co-culture of L929 cells and test materials. Live dead (green/red) staining under 20× objective magnification, FLOID microscope. Microphotographs show cells growing close to and directly under the material.

To assess the effect of the tested materials on cells, cell cultures were performed in their presence. During the study, cell growth was assessed in the immediate vicinity of the material and underneath, at the edge of the material. No changes in cell morphology or other effects on the culture were observed. To confirm the results, staining was performed to assess the number of live and dead cells. The number of dead cells in culture was similar for all test materials and controls. How the test was performed for each material is shown in Figure 4.

#### 2.2.3. Cell Attachment

**Figure 5:**
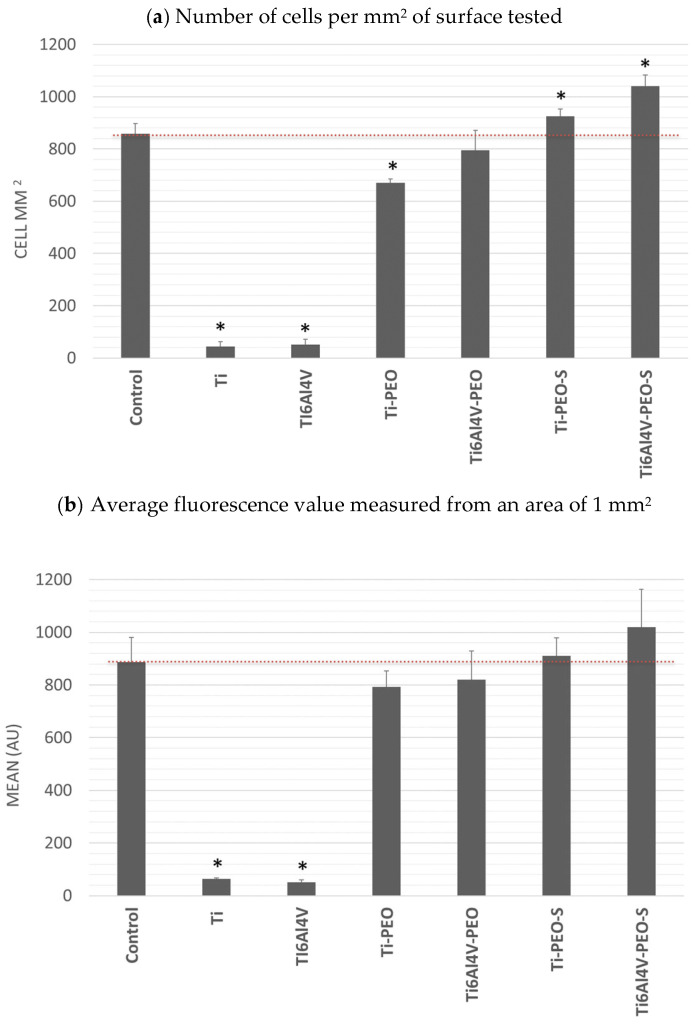
Adhesion and proliferation of NHDF on test surfaces modified after 72 h: (**a**) number of cells per mm^2^ of test surface, (**b**) mean fluorescence value measured from an area of 1 mm^2^. * Statistically significant difference (*p* < 0.05) compared with control.

The most important bioassay performed was the evaluation of cell adhesion and growth directly on the test surfaces. Adherent cells can grow on a variety of surfaces, e.g., glass, but most commonly on surfaces made of different plastics. These surfaces are subjected to various modifications that make the surface in the culture dish more hydrophilic for maximum cell adhesion. In the study conducted, the control surface was modified polystyrene (TPP, Trasadingen, Switzerland). The results obtained are presented as number of cells per test surface and the fluorescence value obtained as the average value of the tested surface. The results shown in Figure 5 indicate that the introduced modifications significantly improved cell adhesion to the test surfaces compared to the original Ti and Ti6Al4V surfaces. For the PEO and PEO-S surfaces, a significant increase in cell number and adhesion fusion is observed, which is similar to or significantly better than the surface standardly used for cell culture, providing optimal cell growth conditions. Comparing PEO and PEO-S surfaces, the latter provides better conditions for cell growth.

### 2.3. Fractal Dimension Analysis

Table 3 presents mean values of fractal dimension in 100 μm × 100 μm scale. The lowest value of FD was seen in Ti6Al4V-PEO (1.782000) and the highest value in Ti6Al4V (1.888920).

Table 4 shows mean values of fractal dimension in 5 μm × 5 μm scale. The lowest value of FD was seen in Ti (1.6931) and the highest value in Ti6Al4V (1.7731).

Table 5 shows Pearson correlation coefficients between FD and Sa and amounts of cells in mm^2^, medium au. We have revealed almost no linear correlation (*r* = −0.02) between Sa and amounts of cells in mm^2^. A very weak negative correlation (*r* = −0.52/*r* = −0.56) was revealed between the value of FD (in scale 100 μm × 100 μm) and amounts of cells in mm^2^, medium au.

### 2.4. Texture Analysis

**Figure 6:**
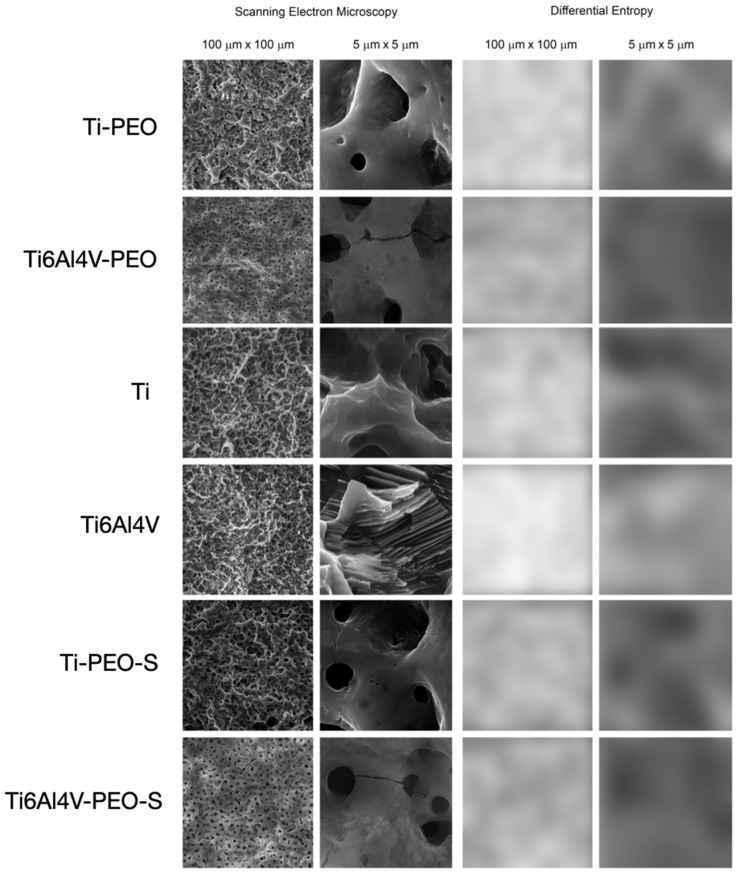
Texture analysis by means of calculation of differential entropy in SEM images on two scales. The two columns on the left show SEM images in the large (100 μm × 100 μm) and small field of view (5 μm × 5 μm). The two columns on the right represent intensity maps of the texture feature studied here in the original SEM image. The whiter areas indicate where the differential entropy is higher (i.e., the surface development is greater), while the darker areas indicate where the differential entropy is low (the implant surface image is more homogeneous). The differences between the tested surfaces are statistically significantly different from one another (*p* < 0.05) in terms of differential entropy (both at low and high magnification).

An example of the results of investigating the surface structure of dental implants by SEM image texture analysis is shown in Figure 6.

When examining the differential entropy as a measure of the development of the implant surface as seen in the SEM, significant differences were noted between the analyzed surfaces in the 100 µm × 100 µm field of view. Statistically significant differences are presented in Table 6.

In turn, when examining the differential entropy of the implant surface image at higher magnification (i.e., in a 5 µm × 5 µm field of view), the texture feature increased, as presented in Table 7.

The increase of Sa was related (*r* = 0.41) with an increase in difference entropy of theimplant surface measured in 100 × 100 µm field of view (Table. 8). DifEntrp (5 µm × 5 µm) was positively correlated with the medium Au and cells [mm^2^] (*r* = 0.72 and *r* = 0.68 respectively). On the contrary, a negative correlation was found between DifEntrp (100 µm × 100 µm) and the medium Au and cells [mm^2^] (*r* = −0.46 and *r* = −0.51 respectively). A moderate correlation (*r* = 0.76) was found between DiffEntrop (100 µm × 100 µm) and FD. No correlation was found between DiffEntrop (5 µm × 5 µm) and FD (*r* = 0.06). Table 8.

Regarding research hypotheses:

1. The first null hypothesis was accepted. None of the experimental samples expressed cytotoxicity.
2. The second null hypothesis has been rejected. Samples after surface treatment have substantially improved cell growth and cell adherence compared to reference SLA samples.
3. The third null hypothesis has been sustained. We did not reveal a correlation between examined features, except a negative correlation between FD, difference entropy (DifEntrp) (in scale 100 μm × 100 μm), and amount of cells, a positive moderate correlation between DifEntrp and number of cells, and a positive strong correlation between the DifEntrp and FD in scale 100 μm × 100 μm.
4. The fourth null hypothesis has been rejected. Our study revealed statistically significant differences between examined surfaces in the aspect of fractal dimension and texture analysis.

## 3. Discussion

The properties of the titanium alloy’s surface, including its microtopography and nanoscale modification and chemical composition, as well as strategies and methods for improving biocompatibility to bone tissue, have been contemporarily well investigated [14,25]. Wang et al. [26] have found that titanium disc surfaces treated with low-temperature argon–oxygen plasma are more hydrophilic compared to nontreated surfaces, and the activation with plasma can enhance the attachment, proliferation, and mineralization of osteoblasts on the surfaces. A recent study by Hadzik et al. [27] has shown that the anodization of Ti-6Al-4V alloy and its further low-pressure radiofrequency oxygen plasma treatment is a promising method of implant transgingival parts modification. Cheng et al. [28] have found that, when bioactive oxide film on titanium dental implants is created by the oxygen plasma, bone cells’ differentiation and osseointegration are improved. Such modifications provide effective binding to hard tissue and, therefore, promote osteointegration. Our study has confirmed that none of the tested surfaces here have any cytotoxic effect on HGF cell lines, so they can be safe when used as dental implant surfaces. Results of our study prove that PEO as well as PEO-S that were treated with a low-pressure OP represent promising options of Ti surface modification. We have reported a significant increase in cell number and adhesion fusion specifically for the tested experimental PEO and PEO-S surfaces compared to the SLA reference surface.

In our study, the fibroblast model was selected to assess the cytocompatibility of the experimental samples. The adhesion behavior of fibroblasts is known to differ among materials with varying degrees of surface roughness. Generally, maximum adhesion is observed for more rough surfaces. The reason for that is the physiology of fibroblasts and their filopodial structure that extends further into grooves and microstructures of rough surfaces. However, a strong positive relationship between bacterial adhesion and plaque accumulation rate and surface roughness in the supragingival region has been also reported. Rough surfaces in such an application may lead to possible peri-implant mucositis and periimplantitis development [29,30,31].

Hence, the ideal micro- and nano-scale titanium surface topography for implants should balance the facility for fibroblast adhesion without simultaneously favoring bacterial growth. One of the methods of titanium surface nanotexturing that matches the abovementioned condition of the ideal surface is electrochemical anodization. Due to that process, the hierarchic superstructure of the TiO_2_ layer in the shape of nanotubes can be synthesized.

It seems that, in the case of the tested experimental surfaces, their cellular response may be related not only to the roughness, which, reaching Sa values from 0.59 to 2.38 nm for various tested surfaces, is within or slightly exceeds the optimal roughness values described in the literature by Wennerberg and Albrektsson, 1–2 µm [5]. Blinova et al. [32] were one of the first to report fibroblast ingrowth both on its surface and inside of it when cultured on a porous titanium implant. They also proved that interactions between the cell and titanium implant are more evident in samples with nonuniform porosity. Moreover, Whiteside et al. [33] reported more specific criteria for biologically advantageous porosity, where surfaces with a greater number of finer pores are favored for cell attachment.

The plasma electrochemical oxidation (PEO) process in a solution containing calcium and phosphorus compounds has to cause the natural oxide layer on the titanium to thicken. EDX study has confirmed the incorporation of calcium and phosphorus into the oxide layer, both in titanium and the Ti6Al4V alloy. Additional treatment of the samples with oxygen plasma did not change the surface morphology of the samples. However, of modified titanium and alloy in the oxide layer, the oxygen content increased slightly—around 1%. The significantly better cell adhesion we achieved compared to reference SLA samples is conditioned by the chemical structure that results from the applied PEO and PEO-S treatment of the implant surfaces.

The fractal dimension analysis (FDA) of the samples has provided a mathematical formalism for describing complex spatial and dynamical structures and describing the entropy potential of the surface. The entropy of the surface generally raises with it complicity and roughness. It has been broadly used in many areas of science, including medicine, dentistry, technology, and materials science [34]. However, the contemporary method is rarely employed to evaluate titanium implant surfaces. In this study, we can observe a negative moderate correlation between FD and amounts of cells in scale 100 µm × 100 µm at a similar level of correlation between difference entropy (DifEntrp) and cell number. This observation has a reflection in FD interpretation. The lower FD’s value is, the more complex of an analyzed surface is present and the higher number of cells we observed. This observation confirmed that FD and DifEntrp in scale 100 µm × 100 µm and DifEntrp in scale 5 µm × 5 µm can be used as surface features in the aspect of cell ability to colonize. Our results show a lack of correlations between Sa and medium Au and cell growth. We revealed a lack of correlation between FD and Sa in contrast to a weak correlation between DifEntrp and Sa in both scales. It is interesting to observe a strong positive correlation between FD and DifEntrp in scale 100 µm × 100 µm in contrast to a weak negative correlation in scale 5 µm × 5 µm. Skośkiewicz-Malinowska et al. [35] reported a positive moderate correlation between the FDA and the number of the fibroblasts when tested on cement surfaces. In our previous study, a weak correlation (*r* = 0.38) between the number of fibroblasts and the fractal dimension in the 100 µm × 100 µm scale was observed. Meanwhile, in the 5 µm × 5 µm scale, the correlation coefficient was lower, and *r* = 0.24 [7].

## 4. Materials and Methods

For the purpose of this study, titanium plates as a sample of different experimental implant surfaces were delivered and tested. The roughness parameters of the surface were measured. Based on the SEM images, fractal dimension analysis and texture analysis was calculated. All the titanium plates were tested for cytotoxicity and, finally, normal human dermal fibroblasts (NHDF) were used to assess the cell culture adhesion to each Ti sample.

### 4.1. Titanium Plates Preparation and Surface Modification

Titanium plates with standard SLA (sandblasted and acid-etched) were prepared and delivered by NanoPrime company (NanoPrime, Dębica, Poland) from the Titanium Grade 4 (Ti) and Titanium Grade 23 (Ti6Al4V). Ti and Ti6Al4V sandblasted and acid-etched Titanium dental implant samples used in this study were previously used in our study as a reference to compare against the Laser-Induced Periodic Surface Structures (LIPSS) dental implant surfaces in our other study [7]. In this study, new experimental electrolytic-modified implant surfaces were used for Ti-PEO and Ti6Al4V-PEO (plasma electrolytic oxidation) and Ti-PEO-S and Ti6Al4V-PEO-S treated with additional low-pressure radio-frequency oxygen plasma (low-pressure RF OP). The details of the titanium surface samples are presented in Table 9.

### 4.2. Surface Analysis Surface Topography Ra, Rz, Sa Measurement

The surface roughness (Sa) parameter was measured with the use of a scanning electron microscope SEM (Thermo Fisher Scientific Inc., Waltham, MA, USA), and 15 keV accelerating voltage was applied. The roughness parameters were measured with Phenom 3D Roughness Reconstruction Software (version 1, Thermo Fisher Scientific Inc., Waltham, MA, USA). Each surface was triplicated, and the measurements were done in four places on each sample. The results were averaged, and a standard deviation (SD) was calculated.

### 4.3. Surface Analysis FDA

We analyzed SEM images under two magnifications: 5000× and 100,000×. Five regions of interest (ROIs) were selected from each image. ROI dimension was 100 μm × 100 μm in case of 5000× magnification and 5 μm × 5 μm for 100,000× magnification. These were applied in the intensity difference fractal dimension counting method to analyze each ROI.

ImageJ version 1.53e (Image Processing and Analysis in Java—Wayne Rasband and contributors, National Institutes of Health, Bethesda, MD, USA, public domain license, https://imagej.nih.gov/ij/, accessed on 1 December 2022) and the FracLac plugin version 2.5 (Charles Sturt University, Bathurs, Australia, public domain license) were applied to do all fractal dimension analyses. The full algorithm of fractal dimension calculation was fully described in our previous study [7].

### 4.4. Surface Analysis TA

MaZda 4.6 (MaZda ver. 4.6, Technical University of Łodź, Institute of Electronics, Łodź, Poland) was used to check how the features were describing analyzed images [36,37]. Primary 8-bit images were reduced to 6 bits. Regions of interest (ROIs) were normalized (μ ± 3σ) to share the same average (μ) and standard deviation (SD) of optical density within the ROI. Difference entropy (DifEntrp) was selected as a texture feature from the co-occurrence matrix to calculate in the ROI:

(1) $$DifEntr=-\sum_{i=1}^{Ng}p_{x-y}\left(i\right)log(p_{x-y}(i))$$

where Σ is sum, *Ng* is the number of levels of optical density in the microphotograph, *i* and *j* are optical density of pixels 5-pixel distant one from another, *p* is probability, and *log* is common logarithm [36,38,39].

### 4.5. Biological Analyses

#### 4.5.1. Cell Culture

In vitro studies were performed using two models: the Normal Human Dermal Fibroblast (NHDF) cell line (Lonza Group, Basel, Switzerland) and L929 cells, (Sigma-Aldrich, Merck Group, Darmstadt, Germany) (The European Collection of Authenticated Cell Cultures-ECACC). Cells were cultured under standard conditions at 37 °C, 5% CO_2_, 95% humidity, in a CO_2_ incubator. Cells were always cultured for a minimum of 2 weeks after thawing before starting a series of experiments. Cell cultures were passaged with trypsin/EDTA solution. Cells were counted using a NucleoCounter^®^ NC-200 automatic cell counter (ChemoMetec A/S, Allerod, Denmark). Cells were cultured in Dulbecco’s modified Eagle medium (DMEM), 10% fetal bovine serum (FBS), penicillin (10,000 U/mL), streptomycin (10 mg/mL), and L-glutamine (200 mM). All culture reagents were purchased from Biological Industries (Biological Industries, Kibbutz Beit-Haemek, Israel).

#### 4.5.2. Preparation of Samples

The test samples were packaged and autoclaved for sterilization. For the in vitro biological cytotoxicity assessment test, liquid extracts of the test materials were prepared according to the provisions of the standard: EN ISO 10993-5: Biological evaluation of medical devices—Part 5: In vitro cytotoxicity testing. Extraction was carried out in sterile, chemically inert, sealed tubes for 24 h at 37 °C in an incubator. Before the experiments involving direct assessment of the interaction of materials with cells, the prepared sections were wetted with serum culture medium. The test material was incubated in the presence of culture medium at a ratio of 1:10. The resulting extract was sterilized by phytoextraction through a 0.22 μm filter.

#### 4.5.3. Evaluation of the Effects on Growth and Vitality of Cell Cultures

The test was performed according to the guidelines of the standard for testing the cytotoxicity of biomaterials. Cells were obtained from culture NHDF. Tests were performed in 96-well plates at 1 × 10^4^ cells/well. Cells were incubated for 24 h (5% CO_2_, 37 °C, 90% humidity) so that the cells formed a monolayer on the plate surface. Before the experiment, each plate was checked under a phase-contrast microscope to ensure that cell growth was relatively uniform across the test plate. After 24 h of incubation, the medium was removed from above the cells. Then, 100 µL of medium containing the appropriate sample extracts, control, or blank only was added to each well. The test plates were incubated for a further 24 h (5% CO_2_, 37 °C, 90% humidity). After 24 h of incubation, each plate was viewed under a phase-contrast microscope to assess the growth of control and extract-treated cells. The observed changes in cell morphology may have been due to the cytotoxic effect of the test sample extract. The culture medium was then carefully removed from the plates and 50 µL of 1 mg/mL MTT solution was added to each well. The plates were incubated for a further 2 h in an incubator at 37 °C. After this time, the MTT solution was removed and 100 µL of isopropanol was added to each well. Absorbance was read on a MultiscanGo reader (Thermo Scientific, Waltham, MA, USA) at 570 nm.

#### 4.5.4. Co-Culture of Cells with Materials

In this assay, L929 cells were seeded into 24-well plates alongside previously placed sterile test biomaterials. In this method, the direct interaction between the cells and the test material was checked by assessing the morphology of the cells and the percentage of live and dead cells (Cell Viability Imaging Kit, Green/Red), which was evaluated on a fluorescence microscope EVOS FLoid (Thermo Scientific, Waltham, MA, USA).

#### 4.5.5. Cell Attachment

Cells for the study were obtained according to the methodology described above. Cultures of normal human dermal fibroblasts (NHDF) were used for the study. The cell suspension prepared for the test was counted and suspended in a culture medium. The density of the cell suspension for the adhesion assay was 1 × 10^6^ cells/mL, and 24-well plates were used for the assay. Materials for the test were placed in wells. Cells in the assay were applied to the wetted material using an automatic pipette. After application, the cells were incubated for 2 h (5% CO_2_, 37 °C, 90% humidity) to allow the cells to adhere to the test materials. After this time, the wells were replenished with serum culture medium in a volume of 1000 µL. The test plates were incubated for a further 72 h (5% CO_2_, 37 °C, 90% humidity). After the incubation time, cells growing on the test surfaces were stained using the Cell Viability Imaging Kit (Blue/Green). Staining involves adding dye to the culture and incubating for 5 to 30 min. After this time, images were taken using a BioTek Lionheart microscope (Agilent Technologies, Santa Clara, CA, USA) using fluorescence excitation with an led illuminator: ex 377 em 447 and ex 469 em 525. Further analysis was performed using GEN5 dedicated image analysis software (Agilent Technologies, Santa Clara, CA, USA). The fluorescence intensity and the number of cells stained with each dye were analyzed. Cells showing blue fluorescence were counted as alive, and green cells as dead.

### 4.6. Statistical Analysis

Statistica version 13.3 (StatSoft, Cracow, Poland) was applied to calculate all fractal dimension analysis statistical tests. The statistically significant level was set to 0.05. The normality of distribution was confirmed by the Shapiro–Wilk test. Due to normal distribution, we performed parametric tests. Analysis of variance (ANOVA) and post hoc least significant difference was used to show differences in fractal dimensions between each surface in two scales. The correlation matrix was applied to calculate the Pearson correlation coefficient (*r*) between the fractal dimension of lesions in two scales and Sa, the number of cells in mm^2^, medium Au, and Sa vs. amounts of cells in mm^2^ and medium Au. The following are the ranges of the r value: *r* greater than or equal to 0.7—strong correlation; *r* between 0.7 and 0.5—moderate; and *r* lower than 0.5—weak correlation. Sample size was calculated on the basis of a power of test. In this study, we used a one way ANOVA for five groups. In this case, the 80% of power of test is achieved for *N* = 50 in each group.

For texture analysis, between-group comparisons were performed with the one way ANOVA or the Kruskal–Wallis test, depending on the presence of normal distribution. Statgraphics Centurion version 18.1.12 (StatPoint Technologies Inc., Warrenton, VA, USA) was used for statistical analyses.

### 4.7. Study Limitations

A limitation of the study was that a machined titanium sample was not used as a control for the cell culture tests. Furthermore, the wettability and microhardness properties of the modified surfaces should be investigated in the future.

## 5. Conclusions

The presented study shows that the surface modification by PEO and PEO-S did not affect the sample cytotoxicity. Greater cell growth of HGF cells was observed on PEO and PEO-S samples compared to reference SLA titanium. The number of cells is correlated with the value of fractal dimension and DifEntrp in scale 100 µm × 100 µm. These two parameters can be used to describe the potential of the surface in the aspect of the ability of a cell to grow. A strong positive correlation between fractal dimension value and DifEntrp was found. In the case of our samples, we have not found a correlation between Sa value and cell growth.

## Figures and Tables

**Table 1:** The semi-quantitative EDX analysis of treated samples, composition in percent (%).

	Ti	Al	V	O ^*^	Ca	P
Ti-PEO	35	-	-	60	3	2
Ti6Al4V-PEO	23	2	1	58	12	3
Ti-PEO-S	35	-	-	61	3	1
Ti6Al4V-PEO-S	21	2	1	61	12	3

* the values must be regarded as only informative.

**Table 2:** Mean values of surface roughness (Sa) for each examined surface in µm.

Surface Name	Sa	SD
Ti-PEO	2.38	0.14
Ti6Al4V-PEO	1.74	0.21
Ti-PEO-S	0.86	0.02
Ti6Al4V-PEO-S	0.59	0.02
TI6Al4V	0.72	0.01
Ti	1.42	0.01

SD—standard deviation.

**Table 3:** Post hoc ANOVA results (least significant difference) for comparison of fractal dimension for ROI size 100 μm × μm between each examined surface.

	Surface Name	FD (ROI = 100 μm × 100 μm)		*p* < 0.05
		Mean	SD	
1	Ti-PEO	1.854240	0.004839	2,3,4,5,6
2	Ti6Al4V-PEO	1.782000	0.007372	1,3,5,6
3	Ti-PEO-S	1.816000	0.014739	1,2,3,5
4	Ti6Al4V-PEO-S	1.782740	0.011618	1,3,5,6
5	Ti	1.804660	0.007630	1,2,4,6
6	Ti6Al4V	1.888920	0.008397	1,2,3,4,5

fractal dimension (FD), standard deviation (SD).

**Table 4:** Post hoc ANOVA results (least significant difference) for comparison of fractal dimension for ROI size 5 μm × μm between each examined surface.

	Surface	FD (ROI = 5 μm × 5 μm)		*p* < 0.05
		Mean	SD	
1	Ti-PEO	1.775360	0.012263	2,3,4,5
2	Ti6Al4V-PEO	1.746280	0.019934	1,3,5,6
3	Ti-PEO-S	1.728280	0.010314	1,2,4,5,6
4	Ti6Al4V-PEO-S	1.751200	0.009727	1,3,5,6
5	Ti	1.693080	0.006080	1,2,3,4,6
6	Ti6Al4V	1.773160	0.016584	2,3,4,5

fractal dimension (FD), standard deviation (SD).

**Table 5:** The values of the Pearson correlation coefficient between the value of fractal dimension calculated in different scale (100 μm × 100 μm and 5 μm × 5μm) and the Sa and number of cells per mm^2^ and medium Au and differential entropy.

Feature	Versus (vs.)	Feature	*r*
FD (100 μm × 100 μm)	vs.	Sa	0.045
FD (5 μm × 5 μm)	vs.	Sa	0.126
FD (100 μm ×100 μm)	vs.	cells [mm^2^]	−0.561
FD (5 μm × 5 μm)	vs.	cells [mm^2^]	0.194
FD (100 μm ×100 μm)	vs.	medium Au	−0.523
FD (5 μm × 5 μm)	vs.	medium Au	0.239
Sa	vs.	cells [mm^2^]	−0.028
Sa	vs.	medium Au	0.084

Pearson correlation coefficient (*r*), fractal dimension (FD), differential entropy (DifEntrp).

**Table 6:** Post hoc ANOVA results (least significant difference) for comparison of difference entropy (DifEntrp) for ROI size 100 μm × μm between each examined surface (SD—standard deviation).

	Surface	DifEntrp		*p* < 0.05
		Mean	SD	
1	Ti-PEO	1.2948	0.0070	3,4,5,6
2	Ti6Al4V-PEO	1.2807	0.0134	4,5,6
3	Ti-PEO-S	1.2577	0.0144	1,4,6
4	Ti6Al4V-PEO-S	1.2208	0.0183	1,2,3,5,6
5	Ti	1.2504	0.0062	1,2,4,6
6	Ti6Al4V	1.3252	0.0055	1,2,3,4,5

difference entropy (DifEntrp), standard deviation (SD).

**Table 7:** Post hoc ANOVA results (least significant difference) for comparison of difference entropy for ROI size 5 μm × 5 μm between each examined surface.

	Surface	DifEntrp		*p* < 0.05
		Mean	SD	
1	Ti-PEO	1.1799	0.0521	5,6
2	Ti6Al4V-PEO	1.2329	0.2890	3,5,6
3	Ti-PEO-S	1.1340	0.0275	2
4	Ti6Al4V-PEO-S	1.1779	0.0326	6
5	Ti	1.1180	0.0738	1,2
6	Ti6Al4V	1.0897	0.0286	1,2,4

difference entropy (DifEntrp), standard deviation (SD).

**Table 8:** The values of the Pearson correlation coefficient (*r*) between the value of difference entropy (DifEntrp) calculated in different scales (100 μm × 100 μm and 5 μm × 5μm) and the Sa and number of cells per mm^2^ and medium Au. No statistically significant relations were found.

Feature	vs.	Feature	*r*
DifEntrp (100 µm × 100 µm)	vs.	Sa	0.2904
DifEntrp (5 µm × 5 µm)	vs.	Sa	0.4173
DifEntrp (100 µm × 100 µm)	vs.	FD	0.7667
DifEntrp (5 µm × 5 µm)	vs.	FD	0.0606
DifEntrp (100 µm × 100 µm)	vs.	cells [mm^2^]	−0.5145
DifEntrp (5 µm × 5 µm)	vs.	cells [mm^2^]	0.6813
DifEntrp (100 µm × 100 µm)	vs.	medium Au	−0.4656
DifEntrp (5 µm × 5 µm)	vs.	medium Au	0.7201

difference entropy (DifEntrp).

**Table 9:** Preparation of the titanium plates.

Name	Titanium Grade	Method of Preparation
Ti	Grade 4	Sandblasted and acid-etched (SLA) Titanium Dental Implant—Al_2_O_3_ sandblasting process with a fraction of 30–100 µm. Purified samples were subjected to the etching process (conditions: oxalic acid 100 g L^−1^, time: 60 min, temperature: boiling). Samples were washed in an ultrasonic cleaner (DEMI water, time: 10 min).
Ti6Al4V	Grade 23	
Ti-PEO	Grade 4	SLA surfaces were anodized in a PEO (plasma electrolytic oxidation) regime. Treatment details were presented in previous studies by Simka et al. [17,18]. An electrolyte was composed of Ca and P compounds. Titanium surfaces were oxidized via the PEO process with a high voltage DC power supply, Kikusui PWR400H, (Kikusui Electronics Corporation, Kanagawa, Japan) at 300 V for 5 min. The PEO treatment was realized via DC galvanostatic anodization (anodic current density = 100 mA cm^−2^) up to limiting voltage. After the process voltage reached the limiting voltage (300 V), the treatment was conducted under a potentiostatic regime. Samples were washed in an ultrasonic cleaner (DEMI water, time: 10 min).
Ti6Al4V-PEO	Grade 23	
Ti-PEO-S	Grade 4	After PEO, samples were treated with low-pressure RF OP and placed in a vacuum chamber for 5 min. Frequency: 40 Mhz, power: 500 Watt. During this time, oxygen was pumped into the chamber (1 L/min).
Ti6Al4V-PEO-S	Grade 23	

## Data Availability

Data available on request.
